# Evolution in an extreme environment: developmental biases and phenotypic integration in the adaptive radiation of antarctic notothenioids

**DOI:** 10.1186/s12862-016-0704-2

**Published:** 2016-06-29

**Authors:** Yinan Hu, Laura Ghigliotti, Marino Vacchi, Eva Pisano, H. William Detrich, R. Craig Albertson

**Affiliations:** Graduate Program in Organismic and Evolutionary Biology, University of Massachusetts, Amherst, MA 01003 USA; Present Address: Department of Biological Sciences, University of Rhode Island, Kingston, RI 02881 USA; Institute of Marine Sciences (ISMAR), CNR, Via De Marini 6, 16149 Genoa, Italy; Department of Earth, Environmental and Life Sciences (DISTAV), University of Genoa, Viale Benedetto XV 5, 16132 Genoa, Italy; Department of Marine and Environmental Sciences, Northeastern University Marine Science Center, Nahant, MA 01908 USA; Department of Biology, University of Massachusetts, Amherst, MA 01003 USA

**Keywords:** Notothenioid, Adaptive radiation, Integration, Evolvability, Channichthyidae

## Abstract

**Background:**

Over the past 40 million years water temperatures have dramatically dropped in the Southern Ocean, which has led to the local extinction of most nearshore fish lineages. The evolution of antifreeze glycoproteins in notothenioids, however, enabled these ancestrally benthic fishes to survive and adapt as temperatures reached the freezing point of seawater (−1.86 °C). Antarctic notothenioids now represent the primary teleost lineage in the Southern Ocean and are of fundamental importance to the local ecosystem. The radiation of notothenioids has been fostered by the evolution of “secondary pelagicism”, the invasion of pelagic habitats, as the group diversified to fill newly available foraging niches in the water column. While elaborate craniofacial modifications have accompanied this adaptive radiation, little is known about how these morphological changes have contributed to the evolutionary success of notothenioids.

**Results:**

We used a 3D-morphometrics approach to investigate patterns of morphological variation in the craniofacial skeleton among notothenioids, and show that variation in head shape is best explained by divergent selection with respect to foraging niche. We document further an accelerated rate of morphological evolution in the icefish family Channichthyidae, and show that their rapid diversification was accompanied by the evolution of relatively high levels of morphological integration. Whereas most studies suggest that extensive integration should constrain phenotypic evolution, icefish stand out as a rare example of increased integration possibly facilitating evolutionary potential. Finally, we show that the unique feeding apparatus in notothenioids in general, and icefish in particular, can be traced to shifts in early developmental patterning mechanisms and ongoing growth of the pharyngeal skeleton.

**Conclusion:**

Our work suggests that ecological opportunity is a major factor driving craniofacial variation in this group. Further, the observation that closely related lineages can differ dramatically in integration suggests that this trait can evolve quickly. We propose that the evolution of high levels of phenotypic integration in icefishes may be considered a key innovation that facilitated their morphological evolution and subsequent ecological expansion.

**Electronic supplementary material:**

The online version of this article (doi:10.1186/s12862-016-0704-2) contains supplementary material, which is available to authorized users.

## Background

Adaptive radiation refers to the rapid diversification of multiple lineages from a common ancestor as a consequence of adaptations to different ecological niches. It is an important evolutionary process that is thought to have produced much of the diversity of life on earth [1, 2]. Research programs in many well-known adaptive radiations, such as Darwin’s finches, East African cichlid fishes, Caribbean *Anolis* lizards, Hawaiian silverswords and more, have made significant contributions towards a better understanding of the processes and mechanisms through which diversity arises and is maintained over time [3–6].

Antarctic notothenioid fishes offer a rare example of an extensive adaptive morphological radiation in an extreme environment [7, 8]. During a series of cooling events over the past 40 million years, the dramatic drop in water temperature of the Southern Ocean has led to the local extinction of most nearshore fish lineages [9]. However, the evolution of antifreeze glycoproteins in notothenioids enabled these ancestrally benthic fishes to survive and adapt as temperatures fell below the freezing point of seawater [10]. The evolution of neutral buoyancy and its associated morphological diversification also fostered their radiation into newly available pelagic foraging niches [8, 11]. Antarctic notothenioids now represent the primary teleost lineage in the waters off of the Antarctic continental shelve, and are of fundamental importance to the local ecosystem [12]. Insights from this clade would complement our current understanding of adaptive radiations in other systems, which largely occur in tropical and subtropical regions.

Adaptive radiations are generally thought to occur via expanded ecological opportunities, which can be facilitated by the evolution of key innovations, extinction of competitors, colonization of new habitats or other scenarios wherein empty niches become available to a lineage [2, 13]. Diversification is then driven by a combination of divergent selection and relaxed stabilizing selection in the new environment. Widely embraced by evolutionary biologists since the modern synthesis, this classic neo-Darwinian view of evolution was built upon the assumption that the phenotypic variation that natural selection acts upon is largely determined by genes, and thus trait evolvability is a direct consequence of additive genetic variation [14, 15]. More recently however, with the emerging field of evo-devo, increasing attention has been devoted to characterizing how phenotypic variation originates in the context of development. It is now widely accepted that development acts to “filter” genetic variation by limiting or biasing patterns of phenotypic variation such that evolution proceeds within the boundaries of developmental constraints [16]. Understanding how these constraints may affect evolvability is considered a central question in the on-going “extended evolutionary synthesis” [17, 18].

The covariation of traits (i.e., phenotypic integration) is predicted to exert profound influences on evolvability (i.e., the potential for systems to evolve), and is an active area of investigation [19–24]. A group of integrated traits is considered a “module”. Within the same module, shifts in one trait are predicted to be accompanied by corresponding changes in all other traits so that the entire module responds to selection in a coordinated fashion and results in a biased phenotypic response. Whether or not such biases are advantageous depends on the specific selection regimes imposed on the phenotype in question. Theoretical work [19] predicts that if selection favors shifts in a subset of traits within a module but not the rest, adaptation may be impeded. Indeed, recent work has consistently found integration to be a constraining force in adaptive evolution [21, 25–27]. Alternatively, if selection happens to align with existing patterns of integration and favors changes within the module as a whole, adaptation could occur rapidly. If correct, this theoretical framework might explain why some lineages exhibit more extensive and/or rapid evolutionary radiations than others. In contrast to integration acting to impede phenotypic evolution, empirical support for integration promoting adaptive diversification is rare. In addition, the extent and speed through which integration itself can evolve across a clade is also not well understood. Additional progress on this front is necessary to further our understanding of trait evolvability.

In this study, we show that variation in head shape aligns well with niche partitioning among notothenioid fishes, highlighting a key role for divergent selection with respect to foraging niche in this group. We also document the evolution of morphological integration among notothenioids, and show that the evolution of highly elevated levels of integration coincides with an accelerated rate of morphological evolution in the icefish family (Channichthyidae). We propose that an elevated magnitude of integration can be considered a key innovation in this group, which may have facilitated their radiation into pelagic feeding habitats. Finally, we trace the unique craniofacial architecture of icefish to discrete shifts in early embryological patterning and subsequent outgrowth of the pharyngeal skeleton. Taken together, we hypothesize that high levels of integration in this group may be a consequence of biases in early craniofacial development that arose as the icefish lineage adapted to a pelagic mode of foraging.

## Methods

### Fish specimen and phylogenetic data

Seventy-eight individuals from 30 notothenioid species were included in the morphological analysis (Additional file 1: Table S1), representing six out of eight notothenioid families [28]. Of these, 29 species among 5 families are Antarctic lineages, while one species, *Eleginops maclovinus*, belongs to the Non-Antarctic lineage Eleginopsidea. We collected 63 individuals of 21 notothenioid species as part of U.S. Antarctic Program project B-037 (H.W.D., Principal Investigator) during cruise LMG14-04 of the ARSV *Laurence M. Gould* in 2014. Most specimens were collected by bottom trawls or via baited fish traps south of Low Island, west of Brabant Island, and in Andvord Bay in the Palmer Archipelago (April – May, 2014). One specimen of *Pogonophryne scotti* was collected by H.W. Detrich (B-037) near Lavoisier Island during cruise LMG12-04 in 2012. Specimens were fixed in 10 formalin on site, and preserved in 70 % ethanol. All specimens were collected in accordance with protocols approved by the Institutional Animal Care and Use Committees (IACUC) of Northeastern University (#12–0306 R). Species were identified by experienced research personnel with reference to Fishes of the Southern Ocean [29], and ambiguities were clarified by sequence analysis on *RPS71* and *myh6*, with reference to published gene sequences [30]. Fourteen specimens of nine notothenioid species from Harvard Museum of Comparative Zoology were also included in the analysis. We used some embryos and larvae from previous collection cruises. *Pleuragramma antarcticum* embryos were collected by M. Vacchi and E. Pisano in the Ross Sea in 2005 [31]. Embryos of *Notothenia coriiceps* were produced by H. W. Detrich at Palmer Station in 2012 via in vitro fertilization. Embryos of *Chaenocephalus aceratus* at ~3 months postfertilization [32] were collected near Brabant Island, Antarctica, on June 27, 2001. Specimens were reared at −1.5 °C in the aquarium at Palmer Station Antarctica and sampled daily for two months.

We used the genetic data and method published in Near et al. (2012) and re-derived the phylogeny of notothenioids because the tree file is not publicly available. In brief, a Bayesian phylogenetic analysis was performed on sequence data that includes five nuclear genes (*RPS71*, *myh6*, *sh3px3*, *tbr1*, and *zic1*) and two mitochondrial genes (*nd2* and *16S rRNA*) from 83 notothenioid species [30]. BEAST analyses were run five times with 3*10^7^ generations each through the CIPRES Science Gateway (Miller et al. 2010), sampling at every 1000 generations. The resulting trees were combined with LogCombiner v2.2.1 (http://beast.bio.ed.ac.uk/LogCombiner), and summarized as a maximum clade credibility tree with node heights set to median divergence ages using TreeAnnotator v2.2.1 (http://beast.bio.ed.ac.uk/TreeAnnotator). The tree file is available as Additional file 2.

### Morphological data collection and analyses

Three-dimensional (3D) landmarks were obtained using the R package StereoMorph [33]. In brief, two cameras were arranged in fixed positions with overlapping fields of view, and calibrated using a standard checkerboard pattern. Landmarks were first digitized on regular 2D images from each camera and then reconstructed into 3D according to the calibration coefficients. Nineteen landmarks were recorded from one side of the head and were then mathematically reflected across the midline assuming left-right symmetry, making a total of 35 landmarks (Additional file 1: Figure S1; Table S2). Head widths at multiple landmark positions were measured to assure the accuracy of reflection. Using routines in the Geomorph package [34], the raw 3D landmark coordinates were aligned with a Generalized Procrustes Analysis. In order to control for common allometric effects across species, shape data were then regressed against centroid size of the head to obtain the residual shape component for subsequent analyses. Feeding habitat and specific diet items were based on published literature (Additional file 1: Table S3). Modes of evolution were evaluated via a multivariate model-fitting approach with the R package mvMORPH [35], using the mean shape for each species. We compared five hypothetical modes of evolution: 1) Brownian Motion model, a random-walk pattern of morphological evolution; 2) Early Burst model, in which most morphological variation was established early in the radiation; 3) Ornstein-Uhlenbeck (OU) single peak model, in which morphological variation was driven by selection towards one evolutionary optimum; 4) OU multi-peak 3 diet model, where morphological variation was driven by selection towards three feeding habitats (benthic, intermediate and pelagic); 5) OU multi-peak 5 diet model, in which the 3 diet model was refined such that both the assumed benthic and pelagic peaks were further partitioned into two separate peaks based on specific food items (Additional file 1: Table S3). Models were ranked according to the Akaike Information Criterion corrected for sample size (AICc) [36]. Raw landmark data is available as Additional file 3.

### Morphological integration analyses

Hypotheses of morphological integration were evaluated with a recently developed method by Adams et al. [37]. In brief, landmarks were divided into hypothetical modules (Additional file 1: Table S2). The degree of covariation between the modules was then evaluated with a partial least squares approach while taking into account phylogenetic relationships. Statistical significance was assessed via phylogenetic permutation with 3000 repeats.

The magnitude of integration, measured as the variance of scaled eigenvalues of partial warp scores (i.e., percent variation explained by each PC axis), was assessed for each species via a recently developed jackknife approach [23]. Integration was first measured for the whole dataset (78 individuals from 30 species) and then re-measured after removing one individual. The difference between the two values provides an indirect measure of integration for that individual, as it represents the relative contribution from that particular individual to the overall magnitude of integration in that group. Average magnitude of integration for each species was then calculated from the individual measures. To account for any potential bias due to uneven sampling between species (i.e., higher sample sizes for some species than others), we repeated the jackknife analysis on a reduced dataset where each species contained only one randomly picked specimen. The results between the two procedures were highly correlated (R-squared: 0.9325, *p*-value: < 2.2e-16), which indicates that our analysis is not sensitive to uneven sampling. In addition, to account for biased phylogenetic sampling (e.g., denser sampling of channichthyids), we repeated the jackknife procedure on further reduced datasets that included one trematomine and one channichthyid species (both randomly picked) only. Finally, we repeated the analysis on a dataset that excluded 5 most closely related channichthyid species (50 % reduction) since this lineage was heavily sampled in our full dataset. In each of these re-analyses, the results were significantly correlated with our original results from the full dataset, and channichthyid species consistently had the highest integration scores. Thus, our method appears to be robust to sampling and phylogenetic relatedness. We report the mean magnitude of integration from the full dataset in the main text (e.g., Figs. 2 and 3). In addition, we provide the raw results from the full and reduced-datasets in Additional file 1: Table S4.

### Morphological disparity and evolutionary rate

Morphological disparity through time was analyzed with the R package Geiger [38] following the methods by Slater et al. [39]. A morphological disparity index (MDI) statistic was derived, which quantifies the difference between the observed disparity profile and the expectation under a null model built from Brownian motion simulations with 10,000 repeats [38, 40]. The most recent 20 % of the tree was discarded to avoid tip over-dispersion, which may overestimate disparity due to incomplete coverage of terminal taxa [40]. Rate of morphological evolution was assessed using routines in the R package Geomorph [34], as described in Adams [41]. All notothenioids were divided into an icefish group and non-icefish group. Evolutionary rate was then calculated according to distances in morphospace between species in each group after phylogenetic transformation. Statistical significance was assessed via randomized phylogenetic simulation with 1000 repeats. It is worth noting that both the disparity and rates analyses are based on a Brownian Motion (BM) model of trait evolution, which may be different from how variation accumulates over time in certain lineages (e.g., OU models could be a better fit). The values returned should therefore be considered conservative.

### Clearing and staining of larval skeletons

To examine patterns of craniofacial development, fish embryos were fixed in 4 % paraformaldehyde, and dehydrated through ethanol series. Skeletal elements were stained based on the protocol described in [42], followed by trypsin digestion (1 % trypsin in a 30 % sodium borate solution) until clear. Samples were then transferred into 80 % glycerol and imaged with a Leica DFC450 C digital microscope camera mounted to a LeicaM165 FC microscope.

### In situ hybridization

*Hand2* (NCBI-GenBank accession no. XM_010780712) riboprobe was made from *Notothenia coriiceps* cDNA using primers that contained T3 (sense) and T7 (antisense) RNA polymerase binding sequences: iHand2_T3F1, CATTAACCCTCACTAAAGGGAACTCAGTAAACGGAGCGGAAG; iHand2_T7R1, TAATACGACTCACTATAGGGTGTCCAGGATTTCCATCAGG. Polymerase recognition sequences are underlined. Whole-mount in situ hybridization was performed as previously described [43]. Notothenioid embryos are extremely hard to obtain, especially those of substrate spawning icefish species that breed at significant depth (>100 m). Thus, in order to obtain pharyngula-stage notothenioid embryos we relied on the pelagic spawning species *Pleuragramma antarcticum*, whose embryos can be obtained in Silverfish Bay (Ross Sea) by sampling through holes drilled in the sea ice, as described in [31].

## Results and discussion

### Divergence in skull shape is correlated with feeding habitat

**Fig. 1:**
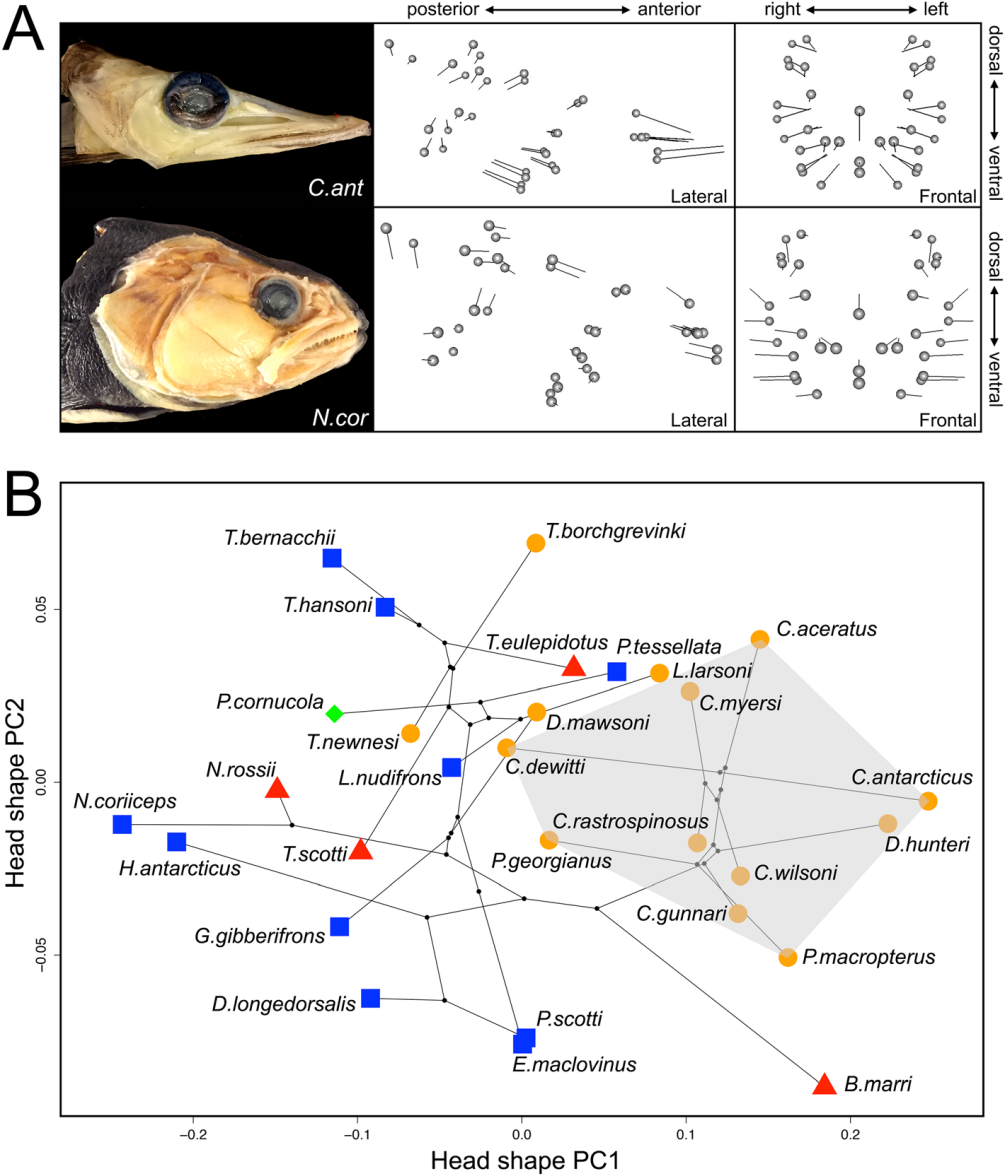
Morphological variation of the head corresponds to niche partitioning among notothenioids. **a** Comparison of head shape between *Cryodraco antarcticus* and *Notothenia coriiceps*, which represent opposite ends of PC1. Ball and stick plot showing the vector displacements of corresponding landmarks from the mean head shape of all notothenioids analyzed in lateral and frontal views. **b** Phylo-morphospace of notothenioids. Shaded area indicates the region occupied by icefish. PC1 explains 64.93 % of the variance, PC2 explains 6.41 %. Species are grouped according to feeding habitats. Blue square: benthic; Red triangle: intermediate; Orange circle: pelagic; Green diamond: unknown

Variation in trophic morphology figures prominently in adaptive radiations because it is directly linked to resource use [13, 44, 45]. In order to investigate patterns of morphological variation in the notothenioid head, we collected 3D shape data from 30 notothenioid species, covering six out of eight families within this clade [28]. We found that the primary axis of shape variation in notothenioids corresponds to their feeding habitat (Fig 1). Species with extreme negative values on PC1 possess wide, robust skulls, short jaws well suited for generating force, and feed predominantly along the bottom of the ocean. In contrast, species with extreme positive values on PC1 have narrow, streamlined skulls, dramatically elongated jaws, and feed mainly on evasive prey items in the water column. Similar ecomorphological shifts were noted by Colombo et al. [46]. Note that this end of the morphospace is largely defined by the white-blooded icefish clade, which is also characterized by a reduction in bone [32, 47, 48] and enhanced buoyancy [8, 9, 49]. These attributes make it energetically feasible for this group to feed in the water column [50, 51].

### Divergent selection in different feeding habitats drives morphological evolution of the notothenioid head

The close association between head shape and diet implies that morphological divergence is being driven, at least in part, by diversifying selection for different feeding habitats. To test this hypothesis, we used a multivariate model-fitting approach to examine the mode of evolution among these fishes (Table 1). The OU multi-peak 3 diet model is the best supported model (Table 1), suggesting divergent selection for feeding habitat has shaped patterns of morphological evolution in this clade. The OU multi-peak 5 diet model was ranked the second highest, thus, both multi-peak models were significantly favored (ΔAICc > 2), providing confidence in the conclusion that ecological opportunity in different feeding habitats was a major factor during the notothenioid radiation. This association between morphological and behavioral divergence represents a characteristic feature of adaptive radiations in vertebrates. Well documented examples include beak size in Darwin’s finches, limb length in *Anolis* lizards, and jaw morphology in cichlid fishes [5, 6, 45, 52]. Notably, the repeated adaptive radiations in African cichlids have resulted in an overall similar pattern of divergence compared to what is observed in notothenioids. In all three rift lakes, the primary axis of head shape variation in cichlids also aligns with a benthic-pelagic spectrum of feeding habitat in spite of two orders of magnitude difference in the age of each radiation [45]. This observation supports the prediction that benthic-pelagic foraging divergence might represent a common selective axis among fish adaptive radiations in general [45].

**Table 1:** Comparison of alternative models of head shape evolution in notothenioids

Model	Log likelihood	AICc	Delta AICc	AICc weight
OU-Diet3	26.1143	-39.50132	0.00	0.693
OU-Diet5	28.42475	-37.24949	2.25	0.225
OU-Single Peak	21.0511	-35.10237	4.40	0.077
Brownian Motion	16.98216	-29.48433	10.02	0.005
Early Burst	16.98212	-26.96424	12.54	0.001

Models are ordered from best to worst based on AICc scores. OU-Diet3: Ornstein-Uhlenbeck (OU) multi-peak model with species assigned to three categories according to feeding habitats (pelagic, intermediate and benthic); OU-Diet5: OU multi-peak model with species assigned to five categories according to feeding habitat and prey items (pelagic-large, pelagic-small, intermediate, benthic-soft, benthic-hard)

### Notothenioids skulls are highly integrated

The first PC axis explained ~65 % of the variance in head shape, while each of the remaining PCs explained less than 7 % of the variance. This indicates that there is a significant amount of correlation among the landmarks examined, and suggests that the entire notothenioid skull may constitute an evolutionary module. To test this hypothesis, we used a partial least squares (PLS) based method to assess the level of covariation between groups of landmarks while controlling for phylogenetic relationships [37]. The strength of covariation was assessed under two hypothesized patterns of modularity. The first is between the anterior and posterior regions of the skull (Additional file 1: Table S2), and represents a functional hypothesis. Whereas the pre-orbital region of the skull is composed largely of the oral jaws and is involved primarily in prey capture, the posterior region is used for feeding and respiration, and houses the brain and sensory organs. Previous work on mammals and fish [22, 53] strongly and consistently supports modularity between these regions of the skull. The second model compares the dorsal and ventral regions of the skull (Additional file 1: Table S2), and represents a developmental hypothesis. Whereas the dorsal portion of the skull is composed primarily of the dermatocranium and develops from both neural crest and non-neural crest mesoderm, the ventral position of the skull is composed largely of the neural crest-derived viscerocranium. Modularity in this dimension has been found in the opercle bones of fishes [25]. Notably, we found no support for modularity in either dimension of the notothenioid skull. Instead, significant levels of covariation were detected between the hypothetical modules (hypothesis 1, anterior-posterior, r_PLS_ = 0.96, *p* < 0.001; hypothesis 2, dorsal-ventral, r_PLS_ = 0.90, *p* < 0.003), indicating that notothenioids possess a highly integrated skull.

**Fig. 2:**
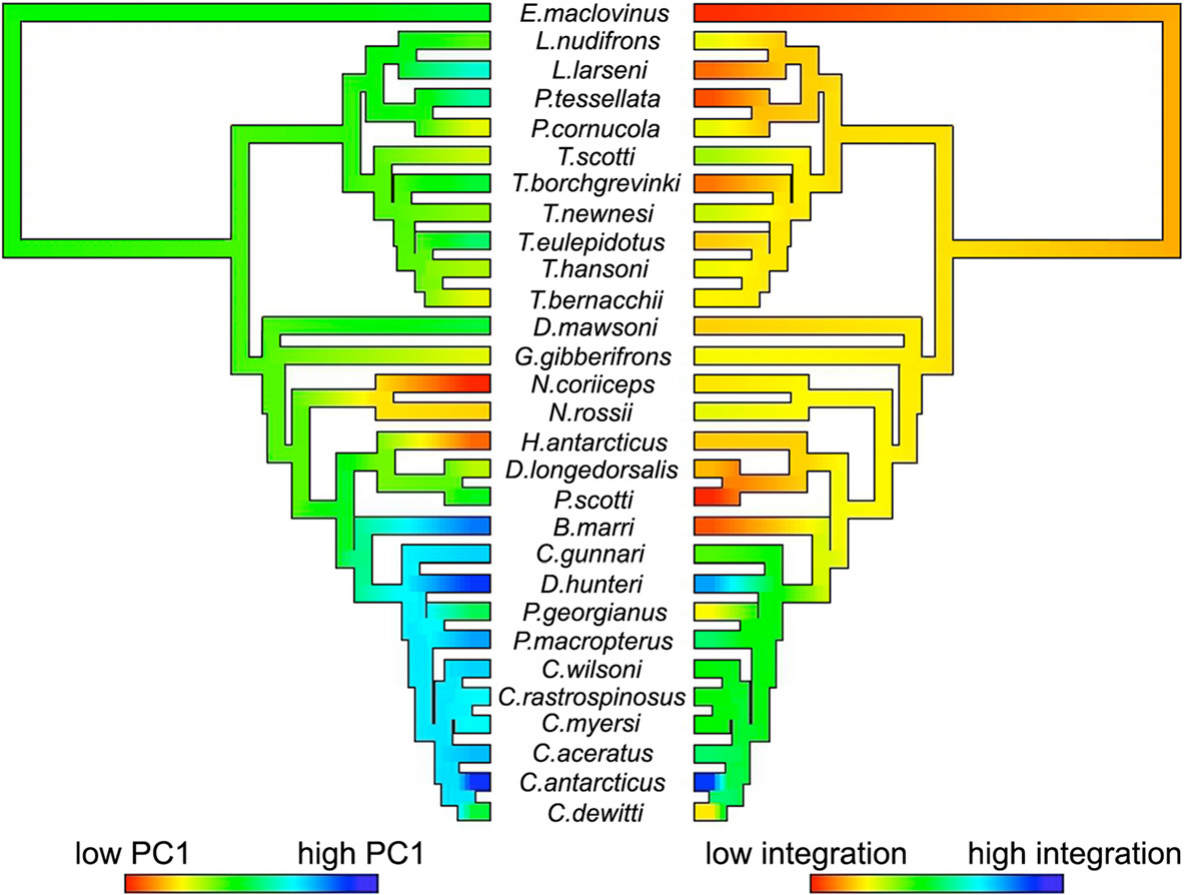
Ancestral state reconstruction of head shape PC1 and integration. Contour map phylogeny shows the estimated evolutionary history of each trait, produced via contMap function in the R package Phytools [81]

**Fig. 3:**
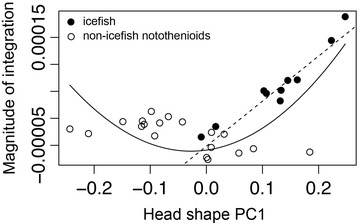
Relationship between head shape PC1 and magnitude of integration. Phylogenetic generalized least squares (PGLS) analysis was performed in R. The expected covariance structure under a Brownian model was first extracted using the corBrownian function from the ape package. Generalized least squares analysis was then carried out using the gls function from the nlme package. Solid curve: quadratic regression across all notothenioids (*p* < 1*10^-4). Dashed line: linear regression within Channichthyidae (*p* < 1*10^-4)

To assess whether high levels of integration in the notothenioid skull are being driven by one or a few lineages, we used a jackknife-based approach to assess the magnitude of integration for each individual [23]. The procedure allowed us to estimate the ancestral state and evaluate the evolution of integration (see for example [54]), as well as its relationship with shape variation across notothenioids. Several notable observations were made based on this analysis (Figs. 2 and 3). First, integration varied widely among closely related notothenioids species, with sister lineages exhibiting alternate extremes of integration. This observation suggests that this trait can evolve over relatively brief time periods. Second, we found that the icefish lineage showed consistently high levels of integration compared to the rest of the notothenioids (*p* < 0.002, *t*-test). Finally, we discerned that morphological integration is correlated with shape (Fig. 3). Across all notothenioids, the best supported relationship between shape (PC1) and integration is quadratic (*p* < 1*10^−4^), such that species with extreme head shapes also exhibit the highest magnitude of integration. This nonlinear relationship between shape and integration has been noted in other lineages [23], and might reflect the inherent relationship between shape and integration when morphology is assessed via a PCA-based method. This is because, by definition, PC1 captures the greatest amount of covariation among phenotypic characters, such that individuals with extreme PC1 scores are expected to contribute more to the overall degree of covariation and thus receive a higher integration score. Nevertheless, despite this statistical caveat, this approach has been shown to be biologically valuable as it measures aspects of morphological variation with genetic underpinnings that are distinct from those determined for standard PCA-based shape [23]. Most relevant to this study, however, is the observation that, when considering the icefish clade alone, a strong linear correlation (Fig. 3) emerges between shape and integration (*p* < 1*10^−4^). The relationship is much stronger than what is observed for the opposite side of the PC axis, underscoring the close relationship between extreme jaw shape and extreme integration in this group. Interestingly, *Bathydraco marri*, a sister species to the channichthyids, showed a very low level of integration, but has evolved a head shape similar to icefish (Figs. 1 and 2), indicating that the tight correlation between integration and head shape is specific to the Channichthyidae. This is especially notable given that nearly half of the notothenioid craniofacial morphospace in our analysis is defined by the icefish lineage, and suggests that the invasion of a pelagic foraging niche may have been facilitated by this shift in integration.

### High magnitude of integration in icefish is associated with elevated shape diversity and accelerated rate of morphological evolution

**Fig. 4:**
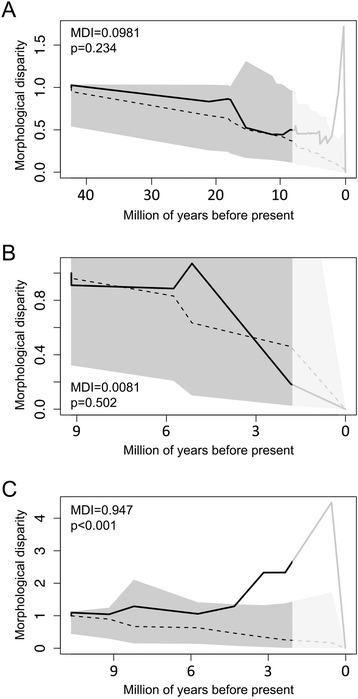
Morphological disparity through time. **a** Disparity plot for all notothenioids, **b** Trematominae, and **c** Channichthyidae. Estimated disparity through time is shown in solid line. Median disparity simulated under Brownian motion condition is shown in dashed line and the grey polygon represents 95 % confidence interval of the simulated disparity. The most recent 20 % of the tree was discarded (masked by light grey in figure) to avoid tip over-dispersion when estimating MDI [40]

We next analyzed morphological disparity through time to evaluate the pattern of morphological diversification among notothenioids (Fig. 4). Morphological disparity across the radiation as a whole is not significantly different from Brownian motion simulations, which suggests a steady increase in diversity over time. We further analyzed disparity in two subclades with decent sampling coverage, Trematominae and Channichthyidae (icefish), and found that while the disparity profile does not deviate from the null model in Trematominae, Channichthyidae exhibited significantly higher levels of disparity, which indicates accelerated rates of morphological diversification within this family. We then compared the rates of evolutionary change in head morphology between channichthyids and the rest of the notothenioids and found that skull shape is evolving at a significantly faster rate in this clade (sigmad.ratio = 1.39, *p* = 0.001). Taken together, these data show that the high level of morphological integration coincides with rapid evolution of skull shape in the Channichthyidae.

### Several innovations underlie the origin and radiation of the notothenioid species flock

The adaptive radiation of Antarctic notothenioids was accompanied (or preceded) by a series of critical innovations [8, 10, 55, 56]. Arguably the most important one was the emergence of antifreeze glycoproteins, which prevent the fish from freezing in the frigid Southern Ocean (at high latitudes, water temperature can remain less than −1.5 °C throughout the year) and is thus critical to their survival in this extreme environment [10]. A second innovation was the evolution of neutral or near-neutral buoyancy [11]. Ancestrally, all notothenioids lacked a functional swim bladder, which is suitable for a benthic lifestyle. However, during the evolution of secondary pelagicism, several lineages have evolved novel mechanisms to gain buoyancy and successfully invade the pelagic foraging niches [8]. To achieve an overall lower density, for example, certain pelagic lineages have evolved enlarged lipid sacs within the axial musculature (i.e., *Pleuragramma antarcticum*), and/or reduced bone mineralization in the skeleton [8, 11, 32, 57]. These novelties compensate for the absence of the swim bladder and significantly reduce the amount of energy required for vertical migration into the water column. It is worth noting that the evolution of antifreeze glycoproteins preceded the ecological diversification of Antarctic notothenioids by 10 million years [30], and reduced skeletal density is also evident in the non-Antarctic species *E. maclovinus* [55]. Thus, these traits may be better viewed as constitutive- or pre-adaptations that enabled survival in this extreme environment and poised the lineage to radiation, but not “key” innovations directly linked to ecological diversification [55]. In contrast, we suggest that the evolution of high magnitudes of integration might be a key innovation unique to icefish, as it accompanied their invasion of a novel foraging niche and concomitant diversification into unique areas of morphospace.

### Integration as a facilitator of evolutionary potential?

A high magnitude of phenotypic integration is generally considered to be a constraint on evolution, because any single mutation would cause corresponding changes in the entire module, resulting in a higher probability of a deleterious outcome. Consistent with this, complex phenotypes, like the craniofacial skeleton, have largely been found to be inherently “modular” [22, 53]. The decoupling of anatomical modules in the vertebrate skull is thought to have facilitated their evolutionary success by enabling distinct functional units to evolve along independent trajectories [22, 53, 58–60]. Alternatively, theory predicts that high levels of integration could also facilitate phenotypic evolution. If the direction of selection coincides with the axis of covariation (i.e., integration), accelerated evolution may result along that direction (i.e., an evolutionary line of least resistance [61–63]). Empirical support for integration in this capacity is comparatively low, and thus Antarctic icefish stand as a rare example of a lineage where relatively high levels of integration are associated with rapid diversification.

### Developmental origins of a unique craniofacial architecture: Roles for patterning and pedomorphism

The Channichthyidae is a unique family of fish [51] that is well-known for their loss of hemoglobin and red-blood cells [64–67], making them the only “white-blooded” vertebrate family on earth. Unlike any other notothenioid subclades, the entire channichthyid family relies heavily on pelagic prey such as krill and fishes, and their mode of prey capture is also unique. Most channichthyid species exhibit a “benthopelagic” mode of foraging wherein they spend much of their time on or close to the ocean floor but venture into the pelagic zone to actively forage on schools of fish and macroinvertebrates. The feeding apparatus of benthopelagic species is characterized by non-protractible, elongate jaws, a wide gape, and many small teeth. This combination of traits allows channichthyids to feed by expanding their buccal cavity, and overtaking large mouthfuls of prey [9].

The expanded ecological niche into the pelagic zone is associated with accelerated lineage diversification in icefish [28, 30]. The exact age of the Channichthyidae remains unclear, mainly because of the lack of a robust fossil record, but estimates range from 3.5 to 20 million years [30, 46, 50]. By any measure, this is a very young family of fish, especially when one considers that development in the frigid Southern Ocean occurs at a very slow pace. It can take up to 6 months for these fishes to hatch, and 5–8 years to reach sexual maturity [51]. Even at the highest estimate of their age, the icefish are on an evolutionary scale similar to the 2–4 million years that characterize many radiations that have taken place in the tropics, where generation times are on the order of 1–2 years. Thus, the evolution of a unique craniofacial architecture in icefish is associated with rapid lineage diversification.

**Fig. 5:**
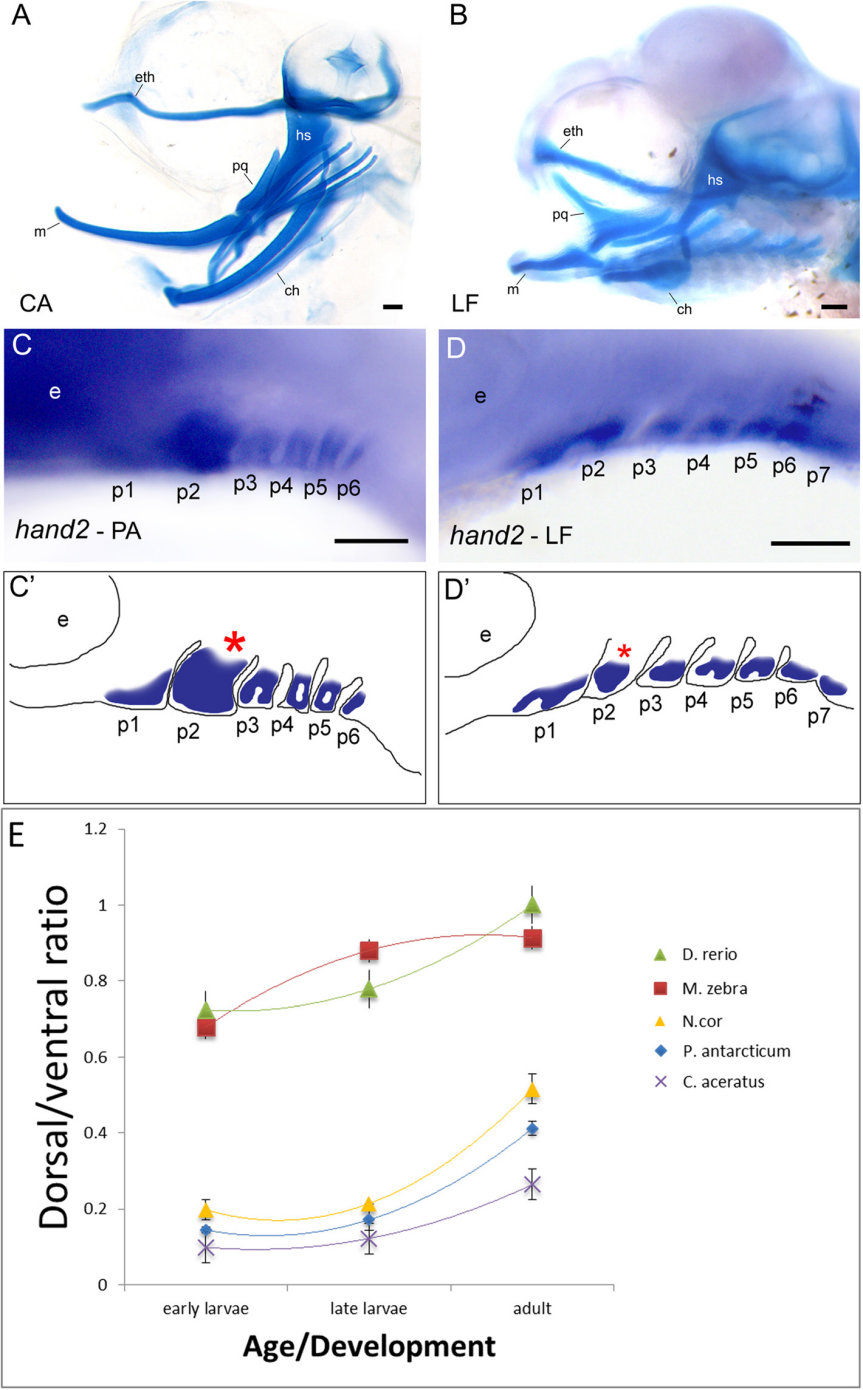
Biased development of anterior-ventral skeleton during early development in notothenioids. **a**-**b** Cleared and stained skeletal preparations. CA: icefish species *C. aceratus*. LF: cichlid species *Labeotropheus fuelleborni*. Note the dramatically enlarged anterior-ventral cartilages in CA compared to LF. ch, ceratohyal; eth, ethmoid plate; hs, hyosymplectic; m, Meckel cartilage; pq, palatoquadrate. We hypothesized that this pattern is key to the highly integrated skull of notothenioids, and that ventral patterning genes may be involved in this change. Differences in *hand2* expression in the developing pharyngeal arches between the pelagic notothenioid species *P. antarcticum* (**c**) and LF (**d**) support this hypothesis. p1–7, pharyngeal arches 1–7. (C’-D’) schematic illustration of *hand2* expression domain in *P. antarcticum* and LF. Note in particular the dorsal expansion of *hand2* expression in the hyoid arch (red asterisk). **e** Comparison of developmental trajectories of pharyngeal cartilages between notothenioids and two other percomorph fishes. Following Kimmel et al. 2007, the dorsal portion of the pharyngeal cartilage was measured as the length of hyosymplectic posterior-dorsal to the interhyal, and the ventral portion was measured as the length of ceratohyal anterior-ventral to the interhyal. Scale bar = 100 μm

We predicted that craniofacial evolution across notothenioids in general, and icefish in particular, might be due to shifts in early developmental patterning events. The vertebrate pharyngeal skeleton is derived from neural crest cells, which migrate into a bilateral series of pharyngeal arches where they condense and differentiate into a conserved set of pharyngeal cartilages. These cartilages are among the first elements of the vertebrate skull to develop, and their developmental patterning occurs along the dorsal-ventral and anterior-posterior axes through a conserved set of regulatory genes [68, 69]. We find that in notothenioids the anterior and ventral cartilages of the pharyngeal skeleton develop earlier and grow more rapidly than in other neoteleost species (Fig. 5 and Additional file 1: Figure S2, also see [32]), which indicates a bias toward the development of anterior and ventral elements of the pharyngeal skeleton early in development. In other fish species, including cichlids and the well-studied zebrafish, the dorsal and ventral elements of the first and second arch appear at approximately the same stage and well after the base of the skull forms [70, 71]. In contrast, the ventral elements of the first and second arch (Meckel’s cartilage and ceratohyal, respectively) in icefish appear in an elongated form well before any other element on the pharyngeal skeleton or skull (Additional file 1: Figure S2). Although this bias is most apparent in *Chaenocephalus aceratus*, a benthopelagic icefish, the relatively short jawed, benthic notothenioid species, *Notothenia coriiceps*, also exhibits a bias toward the development of the ventral elements of the pharyngeal skeleton (Fig. 5e). These data suggest that this unique developmental program may be a synapomorphy of notothenioids.

We show that the expansion of ventral elements in the notothenioid skull can be traced to differential regulation of early developmental patterning mechanisms. Specifically, when we examined the expression of the ventral patterning gene, *hand2*, in cichlid embryos, its expression is limited to the ventral portion of the pharyngeal arches (Fig. 5), as predicted from work in zebrafish [72]. However, in notothenioid embryos the expression domain of this gene is markedly expanded dorsally (Fig. 5). Given the conserved role for this gene in dorsal-ventral patterning of the craniofacial skeleton across vertebrates [72–74], these data are consistent with the hypothesis that shifts in the Edn1/Hand2 regulatory circuit may underlie the development of the unique craniofacial architecture in notothenioids. They also provide empirical support for the idea that this pathway has helped to shape biodiversity in the hyomandibular skeleton in general [75]. How these shifts in the pharyngeal skeleton have influenced the development of the anterior skull (e.g., ethmoid plate), which is also elongated in many notothenioid species, remains an open question. The ethmoid is derived from cranial neural crest cells [76], similar to the pharyngeal skeleton, and thus it is possible that the development of this region of the skull may be influenced by the same genetic pathway as the hyomandibular skeleton (e.g., many zebrafish mutants with defective pharyngeal skeletons also exhibit anterior skull defects [77, 78]).

In addition to an early patterning bias, we find that an expanded ventral skeleton in adult icefish can be explained by the retention of a larval phenotype relative to other notothenioids species. As noted above, all notothenioids examined exhibit an exaggerated ventral skeleton at larval stages. While this ventral bias becomes less obvious over ontogeny in other notothenioids, presumably through differential growth of the dorsal elements, the relative length of ventral elements in adult icefish remains similar to what is observed in the larvae of other notothenioids species (Fig. 5e). Thus, the development of highly elaborated ventral cartilages in larval icefish foreshadows the elongated jaws in adults, providing another example of pedomorphism in this group [32, 47, 48, 56, 79, 80].

With respect to the origins of high levels of integration, we speculate that such dramatic shifts in the early patterning mechanisms of the pharyngeal skeleton could serve to constrain variation across the remainder of the skull. For instance, it is possible that in order to maintain functionality, the development and growth of the icefish craniofacial complex is constrained to accommodate the early and exaggerated development of anterior and ventral elements, which in turn limits variability across other functional units. Thus, extreme jaw elongation via shifts in early developmental patterning events may account for the evolutionary success of the icefish, but as a consequence this mechanism may have led to coordinated variation throughout the rest of the head. To test this hypothesis one could compare early developmental patterning of an icefish species that exhibits high levels of integration and extreme shape along PC1 (e.g., *C. aceratus*) to that in a sister taxon to the icefish clade that still exhibits extreme PC1 values but low magnitudes of craniofacial integration (e.g., *B. marri*).

## Conclusions

Understanding the factors that determine evolvability is an essential component in an ongoing extended evolutionary synthesis [14, 17]. Although theory predicts that phenotypic integration could both limit and promote evolvability [19], empirical studies tend to find integration as a limiting factor to diversification (i.e. an “evolutionary constraint”) [21, 23–26]. In this study, we investigated patterns of morphological diversification in craniofacial skeleton among Antarctic notothenioids. We show that overall these fishes possess a highly integrated skull, and the magnitude of integration is especially high in the icefish family Channichthyidae. We further document an elevated rate of morphological evolution within this clade, which is accompanied by an unexpected tight correlation between integration and shape, indicating that integration might have promoted evolvability among the icefishes. The rapid evolution of head shape among the channichthyids has led to their occupation of a unique region in morphospace, which may have facilitated their invasion into the pelagic feeding habitat. Taken together, this study offers a rare example in which high magnitudes of integration are associated with rapid adaptation and greater evolvability, shedding new light on the mechanisms that influence morphological diversification.

## Abbreviations

AICc, Akaike Information Criterion corrected; BM, Brownian Motion; MDI, Morphological Disparity Index; OU, Ornstein-Uhlenbeck; PC, Principal Component; PCA, Principal Component Analysis; PLS, Partial Least Squares

## Additional file

Additional file 1: Supplemental figures and tables. **Figure S1**. A visualization of the landmarks captured via Stereomorph. **Figure S2**. Pharyngeal skeleton development in the icefish *C. aceratus*. **Table S1**. Feeding habitat and dietary categories for each notothenioid species [9, 12, 29, 82–85]. **Table S2**. List of notothenioid specimens used in morphological analysis. **Table S3**. List of landmarks included in the morphometrics analysis. **Table S4**. Integration analyses on reduced-datasets confirm high magnitude of integration in the Channichthyidae. (PDF 899 kb) Additional file 2: Tree file for notothenioids. (TXT 87.6 kb) Additional file 3: Raw landmark data for 3-D morphometrics, and raw linear measures in larval pharyngeal skeletons. (XLS 243 kb)
