# Supplementary material for: Evolution in an extreme environment: developmental biases and phenotypic integration in the adaptive radiation of antarctic notothenioids
Source: BMC Evol Biol. 2016 Jun 29;16:142. doi: 10.1186/s12862-016-0704-2 (PMC4928320; doi:10.1186/s12862-016-0704-2)
Supplement: Additional file 1: — Supplemental figures and tables. Figure S1. A visualization of the landmarks captured via Stereomorph. Figure S2. Pharyngeal skeleton development in the icefish C. aceratus. Table S1. Feeding habitat and dietary categories for each notothenioid species [9, 12, 29, 82–85]. Table S2. List of notothenioid specimens used in morphological analysis. Table S3. List of landmarks included in the morphometrics analysis. Table S4. Integration analyses on reduced-datasets confirm high magnitude of integration in the Channichthyidae. (PDF 899 kb) [file 12862_2016_704_MOESM1_ESM.pdf]

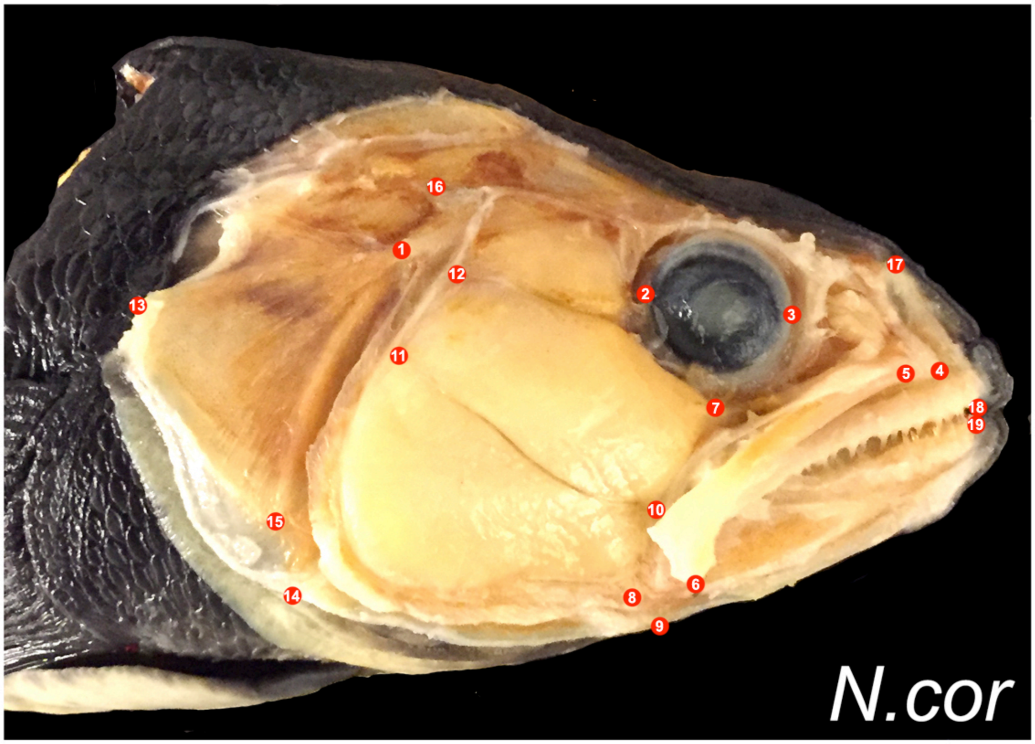

Figure S1

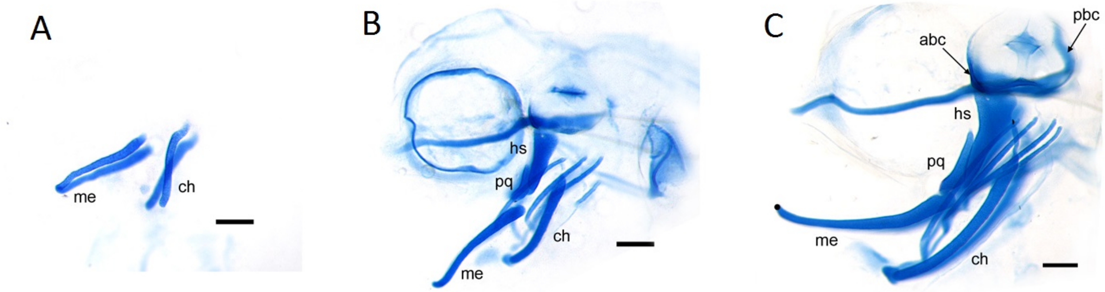

Figure S2

Species name	Number of specimen	Source
Bathydraco_marri	2	B037
Chaenocephalus_aceratus	5	B037
Cryodraco_antarcticus	1	B037
Champsocephalus_gunnari	4	B037
Chionodraco_rastrospinosus	4	B037
Chaenodraco_wilsoni	3	B037
Dolloidraco_longedorsalis	2	B037
Dissostichus_mawsoni	1	B037
Pseudochaenichthys_georgian	2	B037
Gobionotothen_gibberifrons	3	B037
Harpagifer_antarcticus	2	B037
Lepidonotothen kempi	7	B037
Lepidonotothen_larseni	4	B037
Lepidonotothen_nudifrons	4	B037
Notothenia_coriiceps	6	B037
Notothenia_rossii	2	B037
Pagetopsis_macropterus	1	B037
Trematomus_eulepidotus	3	B037
Trematomus_hansoni	3	B037
Trematomus_scotti	3	B037
Pogonophryne_scotti	1	H.W. Detrich 2012
Chionobathyscus_dewitti	1	Harvard MCZ
Chionodraco_myersi	1	Harvard MCZ
Dacodraco_hunteri	2	Harvard MCZ
Eleginops_maclovinus	2	Harvard MCZ
Patagonotothen_tessellata	2	Harvard MCZ
Patagonotothen_cornucola	1	Harvard MCZ
Trematomus_borchgrevinki	2	Harvard MCZ
Trematomus_newnesi	2	Harvard MCZ
Trematomus_bernacchii	2	Harvard MCZ and B037

B037: fish specimen collected during Antarctic expedition B-037 in 2014
Harvard MCZ: specimen from Harvard Museum of Comparative Zoology
H.W. Detrich 2012: specimen collected by H.W.Detrich in 2012

Table S1. List of specimen used in the morphological and integration analysis.

Landmark	Description	Position	Integration hypothesis 1<br>Anterior and posterior module	Integration hypothesis 2<br>Dorsal and Ventral module
1	most anterior-dorsal tip of opercle	Right side	Posterior	Dorsal
2	most posterior point of the eye	Right side	Posterior	Dorsal
3	most anterior point of the eye	Right side	Anterior	Dorsal
4	most anterior-medial point of the maxilla	Right side	Anterior	Dorsal
5	most anterior-lateral corner of the maxilla	Right side	Anterior	Dorsal
6	most posterior-ventral tip of the maxilla	Right side	Anterior	Ventral
7	most anterior-dorsal point of the A1 adductor mandibulae jaw closing muscle	Right side	Anterior	Dorsal
8	quadrate-mandible joint	Right side	Anterior	Ventral
9	insertion of the interopercular ligament on the retroarticular	Right side	Anterior	Ventral
10	most anterior point of the A1-A2 division of adductor mandibulae jaw closing muscle	Right side	Anterior	Ventral
11	most posterior point of the A1-A2 division of adductor mandibulae jaw closing muscle	Right side	Posterior	Ventral
12	most posterior point of the A1-A3 division of adductor mandibulae jaw closing muscle	Right side	Posterior	Dorsal
13	most posterior-dorsal tip of opercle	Right side	Posterior	Dorsal
14	most ventral point of the subopercle-interopercle joint	Right side	Posterior	Ventral
15	most anterior point of the opercle-subopercle joint	Right side	Posterior	Ventral
16	most anterior-dorsal origin of levator operculi muscle	Right side	Posterior	Dorsal
17	most posterior-dorsal tip of the ascending process of the premaxilla	Midline	Anterior	Dorsal
18	most anterior tip of the premaxilla	Midline	Anterior	Dorsal
19	most anterior tip of the dentary	Midline	Anterior	Ventral
20	most anterior-dorsal tip of opercle	Left side	Posterior	Dorsal
21	most posterior point of the eye	Left side	Posterior	Dorsal
22	most anterior point of the eye	Left side	Anterior	Dorsal
23	most anterior-medial point of the maxilla	Left side	Anterior	Dorsal
24	most anterior-lateral corner of the maxilla	Left side	Anterior	Dorsal
25	maxilla-articular joint	Left side	Anterior	Ventral
26	most anterior-dorsal point of the A1 adductor mandibulae jaw closing muscle	Left side	Anterior	Dorsal
27	quadrate-mandible joint	Left side	Anterior	Ventral
28	insertion of the interopercular ligament on the retroarticular	Left side	Anterior	Ventral
29	most anterior point of the A1-A2 division of adductor mandibulae jaw closing muscle	Left side	Anterior	Ventral
30	most posterior point of the A1-A2 division of adductor mandibulae jaw closing muscle	Left side	Posterior	Ventral
31	most posterior point of the A1-A3 division of adductor mandibulae jaw closing muscle	Left side	Posterior	Dorsal
32	most posterior-dorsal tip of opercle	Left side	Posterior	Dorsal
33	most ventral point of the subopercle-interopercle joint	Left side	Posterior	Ventral
34	most anterior point of the opercle-subopercle joint	Left side	Posterior	Ventral
35	most anterior-dorsal origin of levator operculi muscle	Left side	Posterior	Dorsal

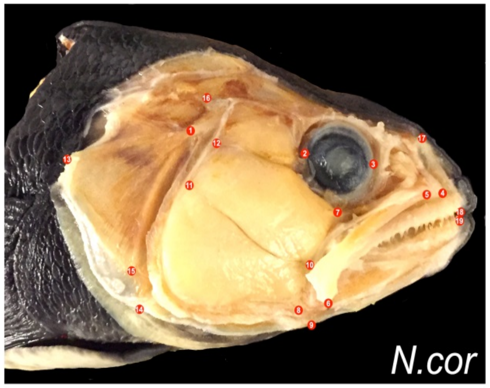

Table S2. Location of landmarks used in this study.

Species name	Integration	PC1	PC2	PC3	Diet3	Diet5
Bathydraco_marri	-0.00006245	0.184272	-0.088188	0.057896	intermediate	intermediate
Chaenocephalus_aceratus	7.02E-05	0.144929	0.041320	-0.032670	pelagic	pelagic-large
Cryodraco_antarcticus	0.000188244	0.247325	-0.005493	-0.001366	pelagic	pelagic-large
Chionobathyscus_dewitti	-0.0000346	-0.009213	0.009988	0.013160	pelagic	pelagic-large
Champsocephalus_gunnari	0.000032275	0.131592	-0.037993	0.023658	pelagic	pelagic-small
Chionodraco_myersi	0.000051	0.102394	0.026290	0.009949	pelagic	pelagic-large
Chionodraco_rastrospinosus	0.0000462	0.106874	-0.017593	-0.016090	pelagic	pelagic-large
Chaenodraco_wilsoni	0.000052	0.133114	-0.027158	-0.004138	pelagic	pelagic-large
Dacodraco_hunteri	0.000144198	0.222818	-0.012011	-0.002645	pelagic	pelagic-large
Dolloidraco_longedorsalis	-0.0000325	-0.092099	-0.062555	0.000278	benthic	benthic-soft
Dissostichus_mawsoni	-0.000026	0.009041	0.020361	0.058994	pelagic	pelagic-large
Eleginops_maclovinus	-0.00007255	0.000426	-0.075828	0.026826	benthic	benthic-soft
Pseudochaenichthys_georgian	-0.000015135	0.016591	-0.016791	-0.012912	pelagic	pelagic-large
Gobionotothen_gibberifrons	-1.11E-05	-0.110953	-0.041779	-0.079664	benthic	benthic-hard
Harpagifer_antarcticus	-0.0000283	-0.210077	-0.017301	-0.046560	benthic	benthic-soft
Lepidonotothen kempi	-1.62E-05	-0.019989	0.030853	-0.025292	benthic	benthic-soft
Lepidonotothen_larseni	-0.000056225	0.083838	0.031539	0.003364	pelagic	pelagic-small
Lepidonotothen_nudifrons	-6.60E-06	-0.042731	0.004286	-0.052951	benthic	benthic-soft
Notothenia_coriiceps	-0.00001969	-0.243217	-0.012239	0.049275	benthic	benthic-hard
Notothenia_rossii	-0.000006104	-0.148882	-0.002411	0.069461	intermediate	intermediate
Patagonotothen_tessellata	-0.0000643	0.057862	0.031956	0.047070	benthic	benthic-soft
Patagonotothen_cornucola	-0.00000487	-0.114030	0.019831	0.009756	UNKNOWN	UNKNOWN
Pagetopsis_macropterus	0.0000713	0.161997	-0.050712	0.001318	pelagic	pelagic-large
Pogonophryne_scotti	-0.0000762	0.002572	-0.074023	0.041543	benthic	benthic-soft
Trematomus_bernacchii	-0.00001485	-0.115506	0.064888	0.000187	benthic	benthic-hard
Trematomus_borchgrevinki	-0.0000539	0.008561	0.069236	0.048176	pelagic	pelagic-small
Trematomus_eulepidotus	-0.0000293	0.031616	0.032835	0.014196	intermediate	intermediate
Trematomus_hansoni	-0.00000852	-0.083203	0.050650	0.031852	benthic	benthic-soft
Trematomus_newnesi	0.00000315	-0.067715	0.014145	-0.037391	pelagic	pelagic-small
Trematomus_scotti	1.28E-05	-0.098088	-0.020230	-0.039998	intermediate	intermediate

pelagic-large: diet includes a considerable proportion of fish
pelagic-small: diet mainly includes small invertebrates
benthic-hard: benthic feeders capable of consuming hard-shelled invertebrates such as clams.
benthic-soft: benthic feeders that feeds on relatively soft preys

References for diet and feeding habitat:

Casaux, R. & Barrera-oro, E., 2013. Dietary overlap in inshore notothenioid fish from the Danco Coast , western Antarctic Peninsula. , 1, pp.1–8.

Eastman, J.T. 1993. Antarctic Fish Biology: Evolution in a Unique Environment. Academic Press, San Diego, 322 pages.

Gon, O. & P.C. Heemstra (eds). 1990. Fishes of the Southern Ocean. J.L.B. Smith Institute of Ichthyology, Grahamstown, 462 pp. 12pls.

Hüne, M. & Vega, R., 2015. Spatial variation in the diet of Patagonotothen tessellata (Pisces, Nototheniidae) from the fjords and channels of southern Chilean Patagonia. Polar Biology.

Licandeo, R.R., Barrientos, C. a. & González, M.T., 2006. Age, growth rates, sex change and feeding habits of notothenioid fish Eleginops maclovinus from the central-southern Chilean coast. Environmental Biology of Fishes, 77(1), pp.51–61.

La Mesa, M., Eastman, J.T. & Licandro, P., 2007. Feeding habits of Bathydraco marri (Pisces, Notothenioidei, Bathydraconidae) from the Ross Sea, Antarctica. Polar Biology, 30(5), pp.541–547.

La Mesa, M., Eastman, J.T. & Vacchi, M., 2004. The role of notothenioid fish in the food web of the Ross Sea shelf waters: a review. Polar Biology, 27(6), pp.321–338.

Table S3. Mean magnitude of morphological integration, principal component scores, feeding habitat and dietary categories for each notothenioid species.

Species name	Full Dataset	Reduced Dataset 1	Reduced Dataset 2	Reduced Dataset 3	Reduced Dataset 4
			Integration values		
Bathydraco_marri	-0.00006245	-0.0003704	-0.000666114	0.00162463	-0.000119338
Chaenocephalus_aceratus	7.02E-05	0.000794457	NA	NA	0.001996142
Cryodraco_antarcticus	0.000188244	0.001090843	NA	NA	NA
Chionobathyscus_dewitti	-0.0000346	-0.000203373	NA	NA	NA
Champsocephalus_gunnari	0.000032275	0.000167494	NA	NA	0.00057438
Chionodraco_myersi	0.000051	0.000297231	0.001378246	NA	NA
Chionodraco_rastrospinosus	0.0000462	0.000128656	NA	NA	NA
Chaenodraco_wilsoni	0.000052	0.000320399	NA	NA	0.000925451
Dacodraco_hunteri	0.000144198	0.000805754	NA	0.006385697	0.002156205
Dolloidraco_longedorsalis	-0.0000325	-9.12E-05	-0.000195075	-0.000452489	-0.000187521
Dissostichus_mawsoni	-0.000026	-0.000140322	-0.000346618	-0.000326051	-0.000204186
Eleginops_maclovinus	-0.00007255	-0.000387386	-0.000611964	-0.001241886	-0.000587792
Pseudochaenichthys_georgian	-0.000015135	-0.000130494	NA	NA	-7.63E-05
Gobionotothen_gibberifrons	-1.11E-05	-6.23E-05	9.18E-05	-0.000314067	-0.000105389
Harpagifer_antarcticus	-0.0000283	-0.000173183	-0.000204714	0.000108444	-0.000228337
Lepidonotothen kempi	-1.62E-05	-0.000212423	-0.000360311	-0.000565507	-0.000286017
Lepidonotothen_larseni	-0.000056225	-0.000321924	-0.000613085	-0.000558986	-0.000406611
Lepidonotothen_nudifrons	-6.60E-06	-7.46E-05	-0.000128819	-0.000633546	-0.000154138
Notothenia_coriiceps	-0.00001969	-0.000179092	-0.000700874	0.000171904	-0.000310506
Notothenia_rossii	-0.000006104	-2.06E-05	-0.0001	-0.00026914	-0.000140784
Patagonotothen_tessellata	-0.0000643	-0.000516879	-0.001041723	-0.001042522	-0.000691366
Patagonotothen_cornucola	-0.00000487	-4.09E-06	-7.76E-05	-0.00050861	-0.000103028
Pagetopsis_macropterus	0.0000713	0.000391006	NA	NA	NA
Pogonophryne_scotti	-0.0000762	-3.75E-04	-0.000912462	-0.00135219	-0.000642979
Trematomus_bernacchii	-0.00001485	-0.000233067	-0.000523446	NA	-0.000408359
Trematomus_borchgrevinki	-0.0000539	-0.000281379	NA	NA	-0.000426933
Trematomus_eulepidotus	-0.0000293	-0.000101557	NA	-0.000486607	-0.00018386
Trematomus_hansoni	-0.00000852	-9.16E-05	NA	NA	-0.00019982
Trematomus_newnesi	0.00000315	-0.000112504	NA	NA	-0.00022207
Trematomus_scotti	1.28E-05	7.94E-05	NA	NA	6.05E-05

Correlation with original dataset					
**r-squared value**	-	0.9301	0.747	0.6917	0.829
**p value**	-	<2.2e-16	9.63E-06	3.95E-05	1.65E-10

Table S4. Integration analyses on reduced-datasets confirm high magnitude of integration in the Channichthyidae.
